# Effect of sarcopenia on hospital stay from post cardiac surgery to discharge

**DOI:** 10.1016/j.ijcha.2022.101003

**Published:** 2022-03-18

**Authors:** Ikuko Shibasaki, Motoshi Ouchi, Taira Fukuda, Go Tsuchiya, Hironaga Ogawa, Yusuke Takei, Masahiro Tezuka, Yuta Kanazawa, Satoshi Katayanagi, Naohiro Nozawa, Takashi Mizushima, Shigeru Toyoda, Hirotsugu Fukuda, Toshiaki Nakajima

**Affiliations:** a Department of Cardiac and Vascular Surgery, Dokkyo Medical University School of Medicine, Shimotsuga, Japan; b Department of Pharmacology and Toxicology, Dokkyo Medical University School of Medicine, Shimotsuga, Tochigi, Japan; c Department of Liberal Arts and Human Development, Kanagawa University of Human Services; d Department of Rehabilitation, Dokkyo Medical University School of Medicine, Shimotsuga, Tochigi, Japan; e Department of Cardiovascular Medicine, Dokkyo Medical University School of Medicine, Shimotsuga, Japan

**Keywords:** Sarcopenia, Hospital stay, Cardiac surgery, ADL, activities of daily living, BMI, body mass index, BNP, brain natriuretic peptide, CPB, cardiopulmonary bypass, CI, confidence interval, CT, computed tomography, eGFR, estimated glomerular filtration rate, ICU, intensive care unit, OR, odds ratio, SMI, skeletal muscle mass index

## Abstract

**Background:**

Cardiovascular surgery in older patients with sarcopenia has high rates of major complications, long hospital stays, readmissions, and discharge transfers. However, the factors that influence the length of hospital stay are unknown. This study aimed was to identify the predictors of the prolonged hospital stay in patients with sarcopenia after cardiovascular surgery.

**Methods:**

A total of 192 patients scheduled for cardiac surgery were enrolled in this retrospective observational study. Sarcopenia was diagnosed preoperatively. Clinical data from the preoperative, intraoperative, and perioperative periods were evaluated to determine the factors influencing the length of hospital stay.

**Results:**

The sarcopenia and non-sarcopenia groups differed significantly in age; body mass index; renal function; intubation time; transfusion of red blood cells; hospital transfer; and hemoglobin, brain natriuretic peptide, and albumin levels. Sarcopenia was the most significant factor influencing prolonged postoperative hospital stay, as well as prolonged intubation time. In the sarcopenia group, albumin levels and cardiopulmonary bypass time were the significant factors affecting hospital stay.

**Conclusions:**

Sarcopenia was the most significant factor associated with prolonged postoperative hospital stay in patients who underwent cardiac surgery. In addition, improving sarcopenia, nutritional status, and shortening cardiopulmonary bypass time appear to shorten the hospital stay in patients with sarcopenia who underwent cardiovascular surgery.

## Highlights

• Sarcopenia was diagnosed in 37.5% (n = 72) patients who underwent open heart surgery.
• The mortality rate for patients who underwent elective surgery was 1.4% (n = 1).
• The median time from surgery to hospital discharge was 20 days.
• Sarcopenia and intubation time affect the length of postoperative hospital stay.
• CPB time affects the length of postoperative hospital stay in sarcopenia.

## 1 Introduction

Globally, Japan has the highest proportion of older people in the total population [1]. The prevalence of sarcopenia has increased with an aging population. The proportion of patients with sarcopenia undergoing heart surgery is increasing [2]. In patients undergoing cardiovascular surgery, the prevalence of sarcopenia before surgery was reported to be 19%–27% [3], [4], [5]. After cardiac surgery, the sarcopenia group reportedly had a longer hospital stay [4]. Moreover, older patients who undergo surgery have greater rates of major complications, prolonged hospital stay, hospital readmissions, and transfer [6], [7], [8].

In cardiovascular surgery, age-related frailty and sarcopenia (loss of muscle mass and strength) have been reported to affect postoperative management [9]. According to the skeletal muscle mass index (SMI) assessed using computed tomography (CT), the prevalence of sarcopenia in men is higher than that in women [10], [11], [12].

Sarcopenia is an important factor affecting postoperative cardiac rehabilitation, slowing the progression of rehabilitation, and affecting hospital transfer [13], [14]. However, data from Japan on the factors that influence prolonged hospital stay in patients with sarcopenia undergoing cardiovascular surgery are lacking. We investigated the physical function, nutritional status, and perioperative data of patients who underwent cardiovascular surgery to identify the predictors of the prolonged hospital stay among patients with sarcopenia.

## 2 Methods

### 2.1 Study population

This was a single-center retrospective study. We consecutively enrolled 192 patients who underwent cardiovascular surgery at Dokkyo Medical University Hospital between October 2015 and October 2020. The enrolled patients were able to walk independently and had no problem with activities of daily living (ADL). ADL were assessed using the Berthel Index method [15] at the time of admission, and the patients were confirmed to have no problems with ADL. Regarding cardiovascular surgery, open-heart surgery with cardiopulmonary bypass (CPB) was performed. We excluded urgent/emergency cases and patients in whom preoperative physical function data used for sarcopenia assessment were missing (cardiac pacemakers, implantable cardioverter defibrillator, metal implants or bolts inserted into the body).

The study protocol was approved by the Dokkyo Medical University Hospital Ethics Committee (approval no: R-30–2). The study was carried out in accordance with the tenets of the Declaration of Helsinki. The need for written informed consent was waived due to the retrospective nature of the study.

Blood samples were collected upon admission. Serum albumin, creatinine, total cholesterol, triglyceride, C-reactive protein, hemoglobin, brain natriuretic peptide (BNP), and hemoglobin A1c levels and estimated glomerular filtration rate (eGFR) were measured.

### 2.2 Sarcopenia diagnostic criteria

SMI, 6-m walking speed, and hand-grip strength were measured to diagnose sarcopenia preoperatively. Sarcopenia was defined according to the Asian Working Group for Sarcopenia criteria (6-m walking speed < 0.8 m/s or hand-grip < 26 kg for men and < 18 kg for women and SMI: <7.0 kg/m2 for men and < 5.7 kg/m2 for women) [16]. Body composition was analyzed in the supine position using a multi-frequency bioelectrical impedance analyzer (InBody S10, Model JMW140, Biospace Co., Ltd., Seoul, Korea) to measure the body fat volume, body fat percentage, and skeletal muscle mass [17]. The SMI was calculated as skeletal muscle mass (kg) /{ height (m)}^2^. The hand-grip strength of the right hand was measured twice, and the higher value was used. The walking speed was measured as the time required to walk 6 m. Patients were classified into sarcopenia and non-sarcopenia groups.

### 2.3 Statistical analysis

Data analysis was performed in three stages. First, the differences in the characteristics between the non-sarcopenia and sarcopenia groups were analyzed using the independent *t*-test (for normally distributed data) and Mann-Whitney *U* test (for non-normally distributed data, non-parametric test) for continuous variables, and χ^2^ test for categorical variables. To compare patient characteristics, factors that affect the period from after surgery to discharge, the groups were categorized according to the median time from surgery to discharge (20 days), and the variables in Table 1 with P-values of < 0.05 were entered into univariate stepwise logistic regression models for sarcopenia. Nine factors with P-values of < 0.05 were entered into the multivariate stepwise logistic regression models (with P < 0.05 for entry). In addition, the sarcopenia group was divided according to the median time from surgery to discharge (25 days), and univariate and multivariate stepwise logistic regression models were applied. We applied a multivariate stepwise logistic regression model (P < 0.05 for entry) for four factors. All the data were analyzed with SPSS software (version 22.0; IBM Corp., Armonk, NY, USA), and P-values of < 0.05 were considered statistically significant.

**Table 1:** Baseline characteristics of the study population.

	N = 192	Median value
Male, n (%)	126 (65.6)	
Age (mean ± SD), years	69.5 ± 10.2	71
BMI (mean ± SD), kg/m^2^	23.4 ± 4.1	22.8
Comorbidity, n (%)		
Hypertension	148 (77.1)	
Diabetes	69 (35.9)	
Dyslipidemia	72 (37.5)	
Hemodialysis	27 (14.1)	
SMI^a^ (mean ± SD), kg/m^2^	6.5 ± 1.3	6.5
6-m walking speed^a^ (mean ± SD), m/s	0.9 ± 0.3	1.0
Hand-grip strength (rt) a (mean ± SD), kg	24.7 ± 8.9	24.5
Albumin (mean ± SD), g/dL	4.0 ± 0.5	4.0
Creatinine (mean ± SD), mg/dL	2.0 ± 2.8	0.9
eGFR < 60 (mean ± SD), mL/min/1.73 m^2^	53.9 ± 27.3	56.7
Total cholesterol^a^ (mean ± SD), mg/dL	167.7 ± 37.6	165
Triglycerides (mean ± SD), mg/dL	106.4 ± 58.3	91
C-reactive protein^a^ (mean ± SD), mg/dL	0.5 ± 1.2	0.12
Hemoglobin (mean ± SD), g/dL	12.5 ± 2.1	12.7
BNP (mean ± SD), pg/mL	311.3 ± 372.4	178.4
Hemoglobin A1c^a^ (mean ± SD), %	6.1 ± 1.1	5.9
Transthoracic echocardiography		
Left ventricular ejection fraction (mean ± SD), %	58.2 ± 12.7	61.0
Type of surgery for cardiovascular disease, n (%)		
Valve replacement/repair	123 (64.1)	
CABG	77 (40.1)	
Aorta replacement	22 (11.5)	
Combined operation	30 (15.6)	
Duration of surgery (mean ± SD), min	363.1 ± 131.6	343
CPB time (mean ± SD), min	185.4 ± 72.8	167
Aorta cross clamp time (mean ± SD), min	120.9 ± 67.3	118
Intraoperative blood loss (mean ± SD), mL	1549.5 ± 1345.9	1276
Transfusion of red blood cells (mean ± SD), unit	7.4 ± 6.3	6
Intubation time (mean ± SD), hours	25.8 ± 58.3	8
ICU stay (mean ± SD), days	2.4 ± 3.3	1
Time from after surgery to discharge (mean ± SD), days	26.0 ± 26.5	20
Discharge destination (home), n (%)	177 (92.1)	
Mortality (mean ± SD), n (%)	1 (0.5)	

SD, standard deviation; BMI, body mass index; SMI, skeletal muscle mass index; eGFR, estimated glomerular filtration rate; BNP, brain natriuretic peptide; CABG, coronary artery bypass grafting; CPB, cardiopulmonary bypass; ICU, intensive care unit.

a Missing values in SMI (n = 6), gait speed (n = 23), handgrip strength (rt) (n = 5), total cholesterol (n = 1), C-reactive protein (n = 1), and hemoglobin A1c (n = 1) were excluded.

## 3 Results

### 3.1 Patient characteristics

**Fig. 1:**
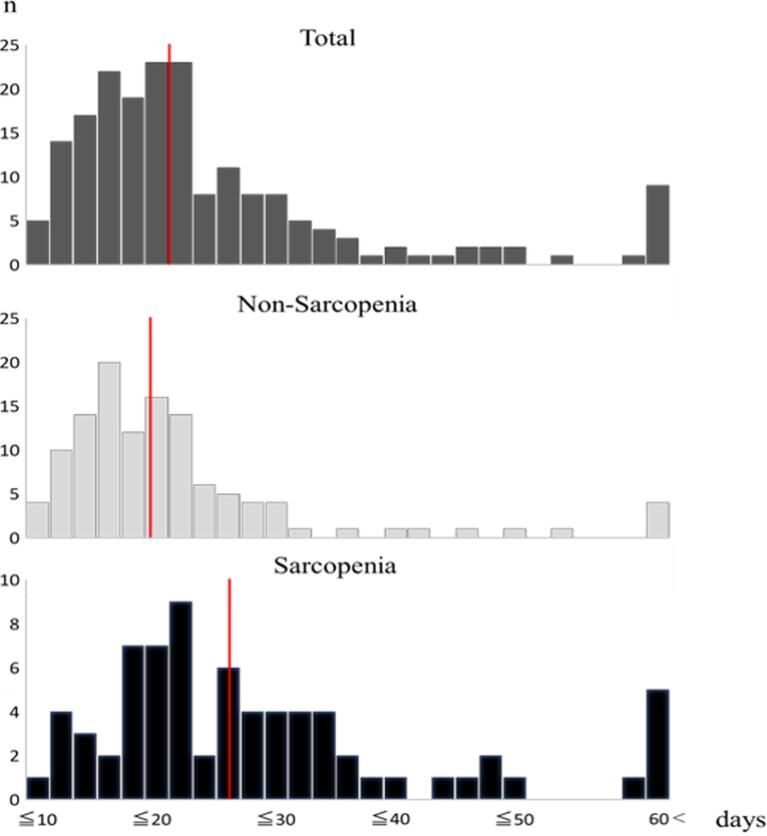
Histograms of the length of hospital stay (days) after cardiac surgery. The red line indicates the median length of hospital stay (days) after cardiac surgery in each group: the left end is ≤ 10 days; the right of end is > 60 days. The median length of hospital stay was 20 days in total, 18.5 days in the non-sarcopenia group, and 25 days in the sarcopenia group. (For interpretation of the references to colour in this figure legend, the reader is referred to the web version of this article.)

The baseline characteristics of the patients (n = 192) are shown in Table 1. In the total study population, 37.5% (n = 72) had sarcopenia. The two groups differed significantly in terms of baseline characteristics (Table 2). Participants in the sarcopenia group were significantly older (73.8 vs. 67.0 years) and had a lower body mass index (BMI; 21.5 vs. 24.5 kg/m^2^) than those in the non-sarcopenia group. In the sarcopenia group, 23.6% of the patients underwent hemodialysis compared with 8.3% in the non-sarcopenia group (P < 0.05). Creatinine and BNP levels were significantly higher and albumin levels, eGFR, and hemoglobin levels were significantly lower in the sarcopenia group than in the non-sarcopenia group (P < 0.05). Regarding the surgical procedures, the proportions of valvular surgery (sarcopenia group, 75.0%; non-sarcopenia group, 57.5%) and aorta replacement/repair surgery (sarcopenia group, 5.6%; non-sarcopenia group, 15.0%) were significantly different between the two groups. Postoperatively, intubation time, time in the intensive care unit (ICU), and time from surgery to discharge were significantly longer in the sarcopenia group than in the sarcopenia group. The discharge home rate was significantly different at 96.7% in the non-sarcopenia group and 84.5% in the sarcopenia group, and the mortality rate was 1.4% in the sarcopenia group. The length of hospital stay from surgery to discharge is shown in Fig. 1. The median number of days was 20 days: 18.5 days in the non-sarcopenia group and 25 days in the sarcopenia group. The mean length of hospital stay was significantly longer in the sarcopenia group than in the non-sarcopenia group (31.6; SD: 33.7 vs. 22.7; SD: 20.5 days, P < 0.001).

**Table 2:** Baseline characteristics of sarcopenia and non-sarcopenia groups.

	Non-sarcopenia group (n = 120)	Sarcopenia group (n = 72)	P-value^a^
Male, n (%)	83 (69.2)	43 (59.7)	0.182
Age (mean ± SD), years	67.0 ± 10.1	73.8 ± 8.8	**≤0.001**
BMI (mean ± SD), kg/m^2^	24.5 ± 4.3	21.5 ± 3.0	**≤0.001**
Comorbidity, n (%)			
Hypertension	95 (79.2)	53 (73.6)	0.375
Diabetes	42 (35)	27 (37.5)	0.727
Dyslipidemia	50 (41.7)	22 (30.6)	0.124
Hemodialysis	10 (8.3)	17 (23.6)	**0.003**
SMI b (mean ± SD), kg/m^2^	6.9 ± 1.2	5.8 ± 1.2	**≤0.001**
6-m walking speed^b^ (mean ± SD), m/s	1.0 ± 0.2	0.8 ± 0.3	**≤0.001**
Hand-grip strength (rt)^b^ (mean ± SD), kg	26.8 ± 8.4	21.3 ± 8.7	**≤0.001**
Albumin (mean ± SD), g/dL	4.4 ± 3.5	3.8 ± 0.5	**≤0.001**
Creatinine (mean ± SD), mg/dL	1.6 ± 2.5	2.6 ± 3.1	**0.010**
eGFR < 60 (mean ± SD), mL/min/1.73 m^2^	58.9 ± 25.1	45.6 ± 30.3	**0.002**
Total cholesterol^b^ (mean ± SD), mg/dL	170.8 ± 37.6	162.5 ± 37.4	0.086
Triglycerides (mean ± SD), mg/dL	112.5 ± 62.3	96.1 ± 49.6	0.076
C-reactive protein^b^ (mean ± SD), mg/dL	0.5 ± 1.3	0.5 ± 1.0	0.202
Hemoglobin (mean ± SD), g/dL	13.0 ± 2.0	11.6 ± 1.6	**≤0.001**
BNP (mean ± SD), pg/mL	233.0 ± 289.7	556.0 ± 807.5	**≤0.001**
Hemoglobin A1c^b^ (mean ± SD), %	6.1 ± 1.0	6.2 ± 1.3	0.583
Transthoracic echocardiography			
Left ventricular ejection fraction (mean ± SD), %	59.4 ± 12.3	56.2 ± 13.3	0.067
Type of surgery for cardiovascular disease, n (%)			
Valve replacement/repair	69 (57.5)	54 (75)	**0.014**
CABG	48 (40)	29 (40.3)	0.970
Aorta replacement	18 (15)	4 (5.6)	**0.047**
Combined operation	15 (12.5)	15 (20.8)	0.124
Duration of surgery (mean ± SD), min	358.1 ± 138.9	371.3 ± 119.0	0.295
CPB time (mean ± SD), min	180.4 ± 76.2	182.0 ± 75.2	0.859
Aorta cross clamp time (mean ± SD), min	120.1 ± 67.7	129.5 ± 68.2	0.563
Intraoperative blood loss (mean ± SD), mL	1462.3 ± 1260.3	1621.0 ± 1066.5	0.196
Transfusion of red blood cells (mean ± SD), unit	6.1 ± 5.7	9.3 ± 5.6	**≤0.001**
Intubation time (mean ± SD), hours	20.9 ± 51.9	23.2 ± 47.5	**0.034**
ICU stay (mean ± SD), days	2.0 ± 2.9	2.5 ± 2.8	**0.032**
Time from after surgery to discharge (mean ± SD), days	22.7 ± 20.5	31.6 ± 33.7	**≤0.001**
Discharge destination (home), n (%)	116 (96.7)	60 (84.5)	**0.003**
Mortality (mean ± SD), n (%)	0 (0)	1 (1.4)	0.196

SD, standard deviation; BMI, body mass index; SMI, skeletal muscle mass index; eGFR, estimated glomerular filtration rate; BNP, brain natriuretic peptide; CABG, coronary artery bypass grafting; CPB, cardiopulmonary bypass; ICU, intensive care unit.

a Using the Chi-squared test or Mann-Whitney *U* test.

b Missing values in SMI (n = 8), gait speed (n = 23), handgrip strength (rt) (n = 5), total cholesterol (n = 1), C-reactive protein (n = 1), and hemoglobin A1c (n = 1) were excluded.

### 3.2 Important factors associated with short- and long-term hospitalization

The median length of hospital stay from surgery to discharge for all patients was 20 days. We performed a multivariate logistic regression analysis with short- and long-term hospitalization as dependent variables to identify independent factors from preoperative, intraoperative, and perioperative data. After adjusting for sarcopenia, age, and sex (Model 1), age and sarcopenia were significant factors (sarcopenia: odds ratio [OR] 2.487; 95% confidence interval [CI] 1.288–4.804, P < 0.01) (Table 3A). In Model 2, we adjusted for Model 1 plus intubation time, BMI, albumin, eGFR < 60 mL/min/1.73 m^2^, hemoglobin, BNP, transfusion of red blood cells, hemoglobin, and ICU stay and found that sarcopenia (OR 2.507; 95% CI 1.138–5.521, P < 0.05) and intubation time (OR 1.027; 95% CI 1.002–1.053, P < 0.05) were significant factors (Table 3B). We analyzed 165 patients excluding hemodialysis patients (165 patients, Supplementary Table 1). Sarcopenia was observed in 55 of the 165 patients (33%). As shown in Supplementary Table 2, multivariate logistic regression analysis showed that sarcopenia and intubation time were the significant factors of short- and long-term hospitalization.

**Table 3:** Factors associated with short-term hospitalization (≤20 days) and long-term hospitalization (>20 days) in multivariate analysis.

A: Model 1					
Independent variable	Odds ratio	95% Confidence interval			P-value^a^
		Lower limit		Upper limit	
Sex^b^	1.148	0.607	–	1.002	0.672
Age^b^	1.035	1.003	–	1.069	**0.034**
Sarcopenia	2.487	1.288	–	4.804	**0.007**
^a^ Using a multivariate logistic regression analysis adjusted for the presence of sarcopenia.^b^Adjusted for sex and age.					

B: Model 2					
Independent variable	Odds ratio	95% Confidence interval			P-value^a^
		Lower limit		Upper limit	
Sex^b^	0.997	0.495	–	2.008	0.994
Age^b^	1.032	0.996	–	1.07	0.081
Sarcopenia	2.507	1.138	–	5.521	**0.023**
Intubation time	1.027	1.002	–	1.053	**0.038**
BMI	1.073	0.985	–	1.169	0.105
Albumin	0.914	0.683	–	1.223	0.546
eGFR<60 mL/min/1.73 m^2^	1.006	0.993	–	1.019	0.381
Hemoglobin	0.87	0.690	–	1.097	0.239
BNP	1.000	0.999	–	1.001	0.963
Transfusion of red blood cells	1.052	0.976	–	1.135	0.187
ICU stay	0.862	0.657	–	1.132	0.285

BMI, body mass index; BNP, brain natriuretic peptide; ICU, intensive care unit.

^a^Using a multivariate logistic regression analysis including each variable with P < 0.05 from Table 1.

^b^Adjusted for sex and age.

## 4 Short- and long-term hospitalization in non-sarcopenia and sarcopenia groups

The comparison of the clinical characteristics between the non-sarcopenia and sarcopenia groups with short-term (<20 days) and long-term (≥20 days) hospitalization is shown in Table 4. In the non-sarcopenia group, the long-term group had a significantly higher hemoglobin A1c level; significantly longer surgery time, CPB time, aortic cross-clamp time, and intubation time; and a significantly higher transfusion rate of red blood cells than the short-term group (all P < 0.05). In the sarcopenia group, the long-term group had a significantly older age and higher BMI; significantly longer surgery time, CPB time, aortic cross-clamp time, and ICU stay; and significantly higher red blood cell transfusions rate than the short-term group (all P < 0.05). Supplementary Table 3 shows the comparison of the clinical characteristics between the non-sarcopenia and sarcopenia groups with short-term (<20 days) and long-term (≥20 days) hospitalization excluding hemodialysis patients. In the sarcopenia group, the long-term group had a higher BMI; concomitant surgery; longer surgery time, CPB time, and aortic cross-clamp time; and higher red blood cell transfusion rate than the short-term group (all P < 0.05).

**Table 4:** Characteristics of the short- and long-term hospitalization groups.

	Non-sarcopenia			Sarcopenia		
	Short-term (<20 days) group	Long-term (≥20 days) group	P-value^a^	Short-term (<20 days) group	Long-term (≥20 days) group	P-value^a^
	(n = 65)	(n = 55)		(n = 19)	(n = 52)	
Male, n (%)	46 (70.8)	37 (67.3)	0.679	13 (68.4)	30 (57.7)	0.413
Age (mean ± SD), years	65.8 ± 10.6	68.4 ± 9.3	0.151	70.9 ± 8.9	75.0 ± 8.7	**0.026**
BMI (mean ± SD), kg/m^2^	24.3 ± 4.2	24.8 ± 4.3	0.606	19.9 ± 1.9	22.1 ± 3.2	**0.003**
Comorbidity, n (%)						
Hypertension	50 (76.9)	45 (81.8)	0.511	14 (73.7)	38 (73.1)	0.959
Diabetes	18 (27.7)	24 (43.6)	0.068	6 (31.6)	21 (40.4)	0.499
Dyslipidemia	26 (40)	24 (43.6)	0.687	5 (26.3)	17 (32.7)	0.607
Hemodialysis	5 (7.7)	5 (9.1)	0.782	4 (21.1)	12 (23.1)	0.857
SMI b (mean ± SD), kg/m^2^	6.8 ± 1.1	6.9 ± 1.3	0.644	6.2 ± 1.5	5.8 ± 1.0	0.201
6-m walking speed b (mean ± SD), m/s	1.0 ± 0.2	1.0 ± 0.2	0.856	0.8 ± 0.3	0.8 ± 0.3	0.870
Hand-grip strength (rt) b (mean ± SD), kg	27.5 ± 8.7	25.9 ± 8.0	0.304	21.8 ± 8.6	21.4 ± 8.7	0.805
Albumin (mean ± SD), g/dL	4.6 ± 4.7	4.0 ± 0.4	0.312	3.9 ± 0.5	3.7 ± 0.5	0.241
Creatinine (mean ± SD), mg/dL	1.7 ± 2.6	1.6 ± 2.4	0.883	2.5 ± 2.8	2.6 ± 3.1	0.948
eGFR < 60 (mean ± SD), mL/min/1.73 m^2^	60.5 ± 26.3	57.0 ± 23.7	0.447	46.9 ± 33.5	45.9 ± 29.1	0.922
Total cholesterol b (mean ± SD), mg/dL	172.2 ± 36.7	169.1 ± 38.9	0.652	158.9 ± 31.6	162.2 ± 38.1	0.659
Triglycerides (mean ± SD), mg/dL	109.7 ± 57.7	115.8 ± 67.6	0.595	102.8 ± 47.0	93.8 ± 51.3	0.360
C-reactive protein b (mean ± SD), mg/dL	0.6 ± 1.6	0.5 ± 0.9	0.696	0.5 ± 1.4	0.5 ± 0.8	0.149
Hemoglobin (mean ± SD), g/dL	13.3 ± 1.6	12.7 ± 2.3	0.113	12.1 ± 2.2	11.5 ± 1.3	0.094
BNP (mean ± SD), pg/mL	226.1 ± 322.7	241.2 ± 247.9	0.772	443.6 ± 475.1	574.1 ± 891.7	0.790
Hemoglobin A1c b (mean ± SD), %	5.9 ± 1.0	6.3 ± 0.9	**0.049**	6.2 ± 1.5	6.2 ± 1.3	0.874
Transthoracic echocardiography						
Left ventricular ejection fraction (mean ± SD), %	60.6 ± 11.9	58.0 ± 12.7	0.419	54.7 ± 13.6	56.7 ± 13.4	0.590
Type of surgery for cardiovascular disease, n (%)						
Valve replacement/repair	38 (58.5)	31 (56.4)	0.817	14 (73.7)	39 (75)	0.910
CABG	24 (36.9)	24 (43.6)	0.454	5 (26.3)	23 (44.2)	0.171
Aorta replacement	9 (13.8)	9 (16.4)	0.700	1 (5.3)	3 (5.8)	0.935
Combined operation	6 (9.2)	9 (16.4)	0.239	1 (5.3)	13 (25)	0.064
Duration of surgery (mean ± SD), min	321.1 ± 93.1	401.9 ± 169.2	**0.001**	306.4 ± 82.7	393.4 ± 122.9	**0.006**
CPB time (mean ± SD), min	160.8 ± 56.3	203.5 ± 89.7	**0.002**	146.4 ± 39.0	193.0 ± 80.9	**0.021**
Aorta cross clamp time (mean ± SD), min	108.3 ± 58.2	133.9 ± 75.6	**0.043**	103.3 ± 41.0	137.5 ± 73.8	**0.031**
Intraoperative blood loss (mean ± SD), mL	1339.8 ± 769.8	1607.0 ± 1660.9	0.276	1268.5 ± 616.8	1753.7 ± 1176.6	0.092
Transfusion of red blood cells (mean ± SD), unit	5.0 ± 4.0	7.5 ± 7.0	**0.016**	6.8 ± 4.9	10.3 ± 5.7	**0.017**
Intubation time (mean ± SD), hours	9.2 ± 8.8	34.7 ± 74.0	**0.007**	13.2 ± 20.7	27.2 ± 54.1	0.175
ICU stay (mean ± SD), days	1.6 ± 1.9	2.5 ± 3.6	0.076	1.5 ± 1.2	2.8 ± 3.2	**0.033**
Time from after surgery to discharge (mean ± SD), days	14.7 ± 2.7	32.1 ± 27.4	**<0.001**	15.5 ± 3.1	37.9 ± 37.8	**<0.001**
Discharge destination (home), n (%)	63 (96.9)	53 (96.4)	0.865	17(89.5)	43(82.7)	0.484

SD, standard deviation; BMI, body mass index; eGFR, estimated glomerular filtration rate; BNP, brain natriuretic peptide; CABG, coronary artery bypass grafting; CPB, cardiopulmonary bypass; ICU, intensive care unit.

One case of in-hospital death in the sarcopenia group was excluded.

a Using the Chi-squared test or Mann-Whitney *U* test.

b Missing values in gait speed (n = 9), handgrip strength (rt) (n = 2), and hemoglobin A1c (n = 1) were excluded.

## 5 Factors associated with long-term hospitalization in patients with sarcopenia

The univariate logistic regression analysis revealed albumin level (OR 0.239; 95% CI 0.082–0.698, P = 0.009), CPB time (OR 1.016; 95% CI 1.006–1.027, P = 0.03), aorta cross-clamp time (OR 1.011; 95% CI 1.002–1.021, P = 0.022), and ICU stay (OR 1.460; 95% CI 1.047–2.037, P = 0.026) as significant risk factors for long-term hospitalization (Table 5). The univariate logistic regression analysis showed albumin level (OR 0.237; 95% CI 0.067–0.844, P = 0.026) and CPB time (OR 1.015; 95% CI 1.003–1.027, P = 0.012) as significant risk factors for long-term hospitalization among non-hemodialysis patients (Supplementary Table 4).

**Table 5:** Factors associated with long-term hospitalization in patients with sarcopenia.

Variables	OR	95% CI			P-value^a^
Albumin	0.239	0.082	-	0.698	**0.009**
CPB time	1.016	1.006	–	1.027	**0.030**
Aorta cross-clamp time	1.011	1.002	–	1.021	**0.022**
ICU stay	1.460	1.047	–	2.037	**0.026**

OR, odds ratio; 95% CI, 95% confidence interval; CPB, cardiopulmonary bypass; ICU, intensive care unit.

One case of in-hospital death in the sarcopenia group was excluded.

The sarcopenia group was divided into two groups based on the median time from after surgery to discharge (25 days). ^a^Using a univariate logistic regression analysis including variables with P < 0.05 in the Chi-square test or Mann-Whitney *U* test.

### 5.1 Independent factors associated with short- and long-term hospitalization in patients with sarcopenia

We conducted a multivariate logistic regression analysis with short- and long-term hospitalization as the dependent variables to identify the independent factors (preoperative, intraoperative, and perioperative data). After adjusting for CPB time, age, and sex (Model 1), the CPB time (OR 1.018; 95% CI 1.007–1.030, P = 0.001) was identified as an independent factor (Table 6A). In Model 2, after adjusting for Model 1 plus albumin, aortic cross-clamp time, and ICU stay, which were significant in the univariate logistic regression analysis, we found that the CPB time was a significant independent factor associated with prolonged hospital stay (OR 1.020; 95% CI 1.002–1.038, P = 0.029) (Table 6B). Similar results were obtained for non-hemodialysis patients (Supplementary Table 5).

**Table 6:** Independent factors of prolonged postoperative hospitalization in patients with sarcopenia in the multivariable analysis.

A: Model 1					
Independent variable	Odds ratio	95% Confidence interval			P-value^a^
		Lower limit		Upper limit	
Sex^b^	1.648	0.555	–	4.892	0.368
Age^b^	1.063	0.996	–	1.134	0.650
CPB time	1.018	1.007	–	1.030	**0.001**
CPB, cardiopulmonary bypass. ^a^Using a multivariate logistic regression analysis adjusted for CPB time. ^b^Adjusted for sex and age.					

B: Model 2					
Independent variable	Odds ratio	95% Confidence interval			P-value^a^
		Lower limit		Upper limit	
Sex^b^	1.899	0.591	–	6.097	0.281
Age^b^	1.071	0.977	–	1.175	0.144
CPB time	1.020	1.002	–	1.038	**0.029**
Albumin	0.302	0.090	–	1.007	0.051
Aorta cross clamp time	0.992	0.976	–	1.009	0.379
ICU stay	1.278	0.926	–	1.764	0.135

CPB, cardiopulmonary bypass; ICU, intensive care unit.

The duration of surgery was excluded because it included CPB time and aorta cross-clamp time.

Concomitant surgery was excluded because it included CABG.

^a^Using a multivariate logistic regression analysis including each variable with P < 0.05 from Table 5.

^b^Adjusted for sex and age.

## 6 Discussion

First, prolonged hospital stay in the sarcopenia group was significantly associated with serum albumin levels and CPB time. The intubation time, ICU stay, and hospital stay from cardiac surgery to discharge were longer in the sarcopenia group than in the non-sarcopenia group. Furthermore, the rate of direct discharge to home was lower. Second, in the sarcopenia group, low levels of hemoglobin, low levels of serum albumin indicating a low-nutrition status, and elevated levels of BNP were observed in the sarcopenia group. In this study, 72 (37.5%) patients were diagnosed with sarcopenia. These patients were older and had impaired physical function compared with those in the non-sarcopenia group. Age-related loss of skeletal muscle mass is associated with low levels of serum albumin indicating a poor nutritional status [18]. However, the serum albumin level was affected by inflammation and infection processes [19], and little evidence supports that it is a marker of sarcopenia. A previous study reported that there is a relationship between high levels of serum albumin and shortened hospital stay [20]. We observed similar results in that low serum albumin levels were associated with prolonged hospital stay.

This study also identified low hemoglobin levels in patients with sarcopenia. Both serum albumin and hemoglobin have been reported to be biomarkers of malnutrition in older people [19]. In another study, sarcopenia (OR 2.4, 95% CI 1.2–4.9), weakness (OR 1.6, 95% CI 1.0–2.5), and slowness (OR 2.0, 95% CI 1.1–3.4) were associated with anemia [21]. In particular, the study reported that low hemoglobin levels have a stronger adverse effect on muscle function (slowness and weakness) than muscle mass. In our study, we identified a relationship between muscle function (hand-grip strength and 6-m walking speed) and anemia in the sarcopenia group (Table 1). By contrast, sex was not a significant factor. Additionally, in the present study, renal dysfunction and elevated levels of BNP were found in the sarcopenia group. The BNP levels of patients with sarcopenia were reportedly higher than those of patients without sarcopenia in chronic heart failure and chronic kidney disease [22]. Furthermore, a previous study reported that the combination of a high sarcopenia score and high BNP level indicated a significantly higher probability of future events [23]. In our study, the BNP levels were significantly higher in the sarcopenia group**;** however, we did not find a significant difference in the left ventricular ejection fraction between the patients with and without sarcopenia. However, further studies on cardiac dysfunction including right ventricular dysfunction are needed. In surgical patients with sarcopenia, sarcopenia generally affects the development of complications in the perioperative period and affects the postoperative prognosis [24], [25], [26]. In the present study, all the patients underwent median sternotomy and CPB which involved invasive surgery and general anesthesia. Moreover, impaired respiratory muscle function due to sarcopenia might affect postoperative complications. Indeed, the intubation time in the sarcopenia group was longer than that in the non-sarcopenia group. Prolonged intubation time was associated with CPB time, age, diabetes, male sex, and ejection fraction, with CPB time being the most strongly correlated variable [27].

Sarcopenia had the most significant effect on the length of hospital stay from after surgery to discharge, followed by prolonged intubation time. Morimoto et al. [13] reported that sarcopenia was the most influential factor in slowing the progress of cardiac rehabilitation and increasing hospital transfer rates. At our hospital, the sarcopenia group had slow cardiac rehabilitation progress. In addition, rehabilitation progress was slow up to the time of walking training.

Rehabilitation nutrition, which combines rehabilitation and nutritional management, is effective for sarcopenia [28]. However, in the current Japanese insurance system, only post-surgical rehabilitation is indicated, and adequate rehabilitation treatment to improve sarcopenia is challenging in short preoperative hospitalization periods. Drudi et al. [29] reported that preoperative exercise training may improve clinical outcomes and physical function in patients undergoing cardiovascular surgery. Waite et al. [30] reported that home preoperative rehabilitation may improve physical function and shorten hospital stay for frail patients undergoing cardiovascular surgery; however, the number of subjects was small (n = 20), and a large randomized controlled trial is needed to determine safety. Nevertheless, several previous studies have shown that “pre-surgical rehabilitation” can lead to good postoperative outcomes [31], [32]. To improve on sarcopenia before surgery, preoperative rehabilitation should be covered by insurance as it could improve physical functions, eliminate psychological factors, shorten hospital stay, improve the home discharge rate, and reduce medical costs.

Finally, CPB time was identified as a factor affecting the length of postoperative hospital stay. Therefore, to shorten the length of hospital stay after cardiovascular surgery, it is necessary to shorten the CPB time.

## 7 Study limitations

The main limitations of this study are the small sample size and retrospective design. Additionally, we excluded urgent/emergency cases and patients in whom preoperative physical function data used for sarcopenia assessment were missing. Thus, there is a possibility that our analysis excluded subjects, which may have affected some data.

## 8 Conclusion

Sarcopenia was the most influential factor of postoperative hospital stay; in addition, preoperative albumin level and CPB time were the independent factors associated with the length of postoperative hospital stay. Further studies are needed to clarify whether preoperative/early postoperative rehabilitation, nutrition, and shortening the artificial CPB time can reduce the length of hospital stay in the sarcopenia group.

## Declaration of Competing Interest

The authors declare that they have no known competing financial interests or personal relationships that could have appeared to influence the work reported in this paper.
