# Supplementary material for: Effect of sarcopenia on hospital stay from post cardiac surgery to discharge
Source: Int J Cardiol Heart Vasc. 2022 Mar 18;39:101003. doi: 10.1016/j.ijcha.2022.101003 (PMC8933819; doi:10.1016/j.ijcha.2022.101003)
Supplement: Supplementary data 1 [file mmc1.docx]

Supplementary Table 1　Baseline characteristics of sarcopenia and non-sarcopenia groups (excluding hemodialysis patients)

|  | Total (n=165) | Median value | Non-sarcopenia group (n=110) | Sarcopenia group (n=55) | P-value^a^ |
| --- | --- | --- | --- | --- | --- |
| Male, n (%) | 126 (65.6) |  | 73 (66.4) | 32 (58.2) | 0.303 |
| Age (mean±SD), years | 69.3±10.2 | 71.0 | 67.1±10.0 | 73.8±9.1 | **≤0.001** |
| BMI (mean±SD), kg/m^2^ | 23.6±4.1 | 23.1 | 24.5±4.2 | 21.7±2.9 | **≤0.001** |
| Comorbidity, n (%) |  |  |  |  |  |
| Hypertension | 124 (75.2) |  | 86 (78.2) | 38 (69.1) | 0.203 |
| Diabetes | 55 (33.3) |  | 34 (30.9) | 21 (38.2) | 0.350 |
| Dyslipidemia | 67 (40.6) |  | 48 (43.6) | 19 (34.5) | 0.262 |
| SMI^b^ (mean±SD), kg/m^2^ | 6.5±1.2 | 6.6 | 6.9±1.1 | 5.9±1.1 | **≤0.001** |
| 6-m walking speed^b^ (mean±SD), m/s | 1.0±0.3 | 0.97 | 1.0±0.2 | 0.8±0.3 | **≤0.001** |
| Hand-grip strength (rt)^b^ (mean±SD), kg | 25.3±8.4 | 24.9 | 26.9±8.2 | 22.1±8.0 | **0.001** |
| Albumin (mean±SD), g/dL | 4.0±0.5 | 4.1 | 4.4±3.6 | 3.8±0.5 | **0.001** |
| Creatinine (mean±SD), mg/dL | 1.0±0.7 | 0.9 | 1.0±0.7 | 1.1±0.7 | 0.274 |
| eGFR<60 (mean±SD), mL/min/1.73 m^2^ | 61.4±21.3 | 61.9 | 63.4±20.6 | 57.8±20.6 | 0.100 |
| Total cholesterol^b^ (mean±SD), mg/dL | 167.8±37.2 | 165.5 | 173.2±37.3 | 157.1±34.7 | **0.005** |
| Triglycerides (mean±SD), mg/dL | 108.1±60.4 | 90.0 | 114.4±63.8 | 95.7±51.3 | 0.055 |
| C-reactive protein^b^ (mean±SD), mg/dL | 0.4±0.9 | 0.09 | 0.4±1.0 | 0.4±1.0 | 0.562 |
| Hemoglobin (mean±SD), g/dL | 12.7±2.1 | 12.8 | 13.1±1.9 | 11.8±1.6 | **≤0.001** |
| BNP (mean±SD), pg/mL | 276.2±311.6 | 171.0 | 218.6±262.2 | 427.1±470.7 | **≤0.001** |
| Hemoglobin A1c^b^ (mean±SD), % | 6.2±1.1 | 6.0 | 6.0±1.0 | 6.4±1.4 | 0.276 |
| Transthoracic echocardiography |  |  |  |  |  |
| Left ventricular ejection fraction (mean±SD), % | 58.5±12.5 | 61.8 | 59.7±12.4 | 56.1±12.6 | 0.057 |
| Type of surgery for cardiovascular disease, n (%) |  |  |  |  |  |
| Valve replacement/repair | 108 (65.5) |  | 66 (60.0) | 42 (76.4) | **0.037** |
| CABG | 62 (37.6) |  | 40 (6.4) | 22 (40.0) | 0.649 |
| Aorta replacement | 20 (12.1) |  | 18 (16.4) | 2 (3.6) | **0.018** |
| Combined operation | 25 (15.2) |  | 11 (12.7) | 11 (20.0) | 0.219 |
| Duration of surgery (mean±SD), min | 364.2±136.7 | 343.0 | 360.7±143.9 | 371.2±122.1 | 0.342 |
| CPB time (mean±SD), min | 187.5±71.9 | 169.0 | 123.9±68.2 | 130.6±71.9 | 0.889 |
| Aorta cross clamp time (mean±SD), min | 121.1±68.0 | 120.0 | 123.9±68.2 | 130.6±71.9 | 0.942 |
| Intraoperative blood loss (mean±SD), mL | 1562.6±1403.5 | 1254.0 | 1478.9±1312.9 | 1567.3±1022.9 | 0.393 |
| Transfusion of red blood cells (mean±SD), unit | 7.0±6.2 | 6.0 | 5.9±5.8 | 8.8±5.2 | **≤0.001** |
| Intubation time (mean±SD), hours | 28.2±61.9 | 7.0 | 21.8±54.1 | 27.5±53.7 | **0.022** |
| ICU stay (mean±SD), days | 2.5±3.4 | 1.0 | 2.1±3.0 | 2.6±3.2 | 0.088 |
| Time from after surgery to discharge (mean±SD), days | 26.3±28.1 | 20.0 | 23.1±21.3 | 32.9±37.6 | **≤0.001** |
| Discharge destination (home), n (%) | 154 (93.3) |  | 106 (96.4) | 48 (87.3) | **0.027** |
| Mortality (mean±SD), n (%) | 0 (0) |  | 0 (0) | 0 (0) | 1.000 |

SD, standard deviation; BMI, body mass index; SMI, skeletal muscle mass index; eGFR, estimated glomerular filtration rate; BNP, brain natriuretic peptide; CABG, coronary artery bypass grafting; CPB, cardiopulmonary bypass; ICU, intensive care unit.

^a^Using the Chi-squared test or Mann-Whitney U test.

^b^Missing values in SMI (n=6), gait speed (n=19), handgrip strength (rt) (n=4), total cholesterol (n=1), and hemoglobin A1c (n=1) were excluded.

Supplementary Table 2. Factors associated with short-term hospitalization (≤20 days) and long-term hospitalization (>20 days) in multivariate analysis (excluding hemodialysis patients

A: Model

| Independent variable | Odds ratio | 95% Confidence interval | | | P-value^a^ |
| --- | --- | --- | --- | --- | --- |
|  |  | Lower limit |  | Upper limit |  |
| Sex^b^ | 0.815 | 0.414 | - | 1.602 | 0.552 |
| Age^b^ | 1.034 | 0.999 | - | 1.070 | 0.059 |
| Sarcopenia | 2.599 | 1.249 | - | 5.405 | **0.011** |

B: Model 2

| Independent variable | Odds ratio | 95% Confidence interval | | | P-value^a^ |
| --- | --- | --- | --- | --- | --- |
|  |  | Lower limit |  | Upper limit |  |
| Sex^b^ | 0.997 | 0.495 | - | 2.008 | 0.917 |
| Age^b^ | 1.032 | 0.996 | - | 1.07 | 0.193 |
| Sarcopenia | 2.507 | 1.138 | - | 5.521 | **0.038** |
| Intubation time | 0.862 | 0.657 | - | 1.132 | **0.047** |
| BMI | 1.073 | 0.985 | - | 1.169 | 0.303 |
| Albumin | 0.914 | 0.683 | - | 1.223 | 0.587 |
| Total cholesterol | 1.006 | 0.993 | - | 1.019 | 0.735 |
| Hemoglobin | 0.87 | 0.69 | - | 1.097 | 0.234 |
| BNP | 1.000 | 0.999 | - | 1.001 | 0.404 |
| Transfusion of red blood cells | 1.052 | 0.976 | - | 1.135 | 0.559 |

Duration of surgery was excluded because it included CPB time and aorta cross clamp time.

^a^Using a multivariate logistic regression analysis with variables with P<0.05 from Supplemental Table 1.

^b^Adjusted for sex and age.

BMI: body mass index; BNP: brain natriuretic peptide.

Supplementary Table 3 Characteristics of the short- and long-term hospitalization groups (excluding hemodialysis patients)

|  | Non-sarcopenia | | | Sarcopenia | | |
| --- | --- | --- | --- | --- | --- | --- |
|  | Short-term (<20 days) group  (n=60) | Long-term (≥20 days) group  (n=50) | P-value^a^ | Short-term (<20 days) group  (n=15) | Long-term (≥20 days) group  (n=40) | P-value^a^ |
| Male, n (%) | 41 (68.3) | 32 (64.0) | 0.632 | 10 (66.7) | 18 (45.0) | 0.435 |
| Age (mean±SD), years | 65.8±10.4 | 68.7±9.4 | 0.119 | 71.1±9.9 | 74.8±8.7 | 0.108 |
| BMI (mean±SD), kg/m^2^ | 24.4±4.2 | 24.8±4.4 | 0.753 | 20.3±1.1 | 22.2±3.2 | **0.020** |
| Comorbidity, n (%) |  |  |  |  |  |  |
| Hypertension | 46 (76.7) | 40 (80.0) | 0.673 | 10 (66.7) | 28 (70.0) | 0.812 |
| Diabetes | 15 (25.0) | 19 (38.0) | 0.142 | 5 (33.3) | 16 (40.0) | 0.650 |
| Dyslipidemia | 26 (43.3) | 22 (44.0) | 0.944 | 4 (26.7) | 15 (37.5) | 0.452 |
| SMI^b^ (mean±SD), kg/m^2^ | 6.9±1.1 | 6.8±1.2 | 0.744 | 6.2±1.5 | 5.8±1.0 | 0.218 |
| 6-m walking speed^b^ (mean±SD), m/s | 1.0±0.2 | 1.0±0.2 | 0.248 | 0.9±0.3 | 0.8±0.3 | 0.657 |
| Hand-grip strength (rt)^b^ (mean±SD), kg | 27.8±8.2 | 25.7±8.2 | 0.398 | 23.0±7.9 | 21.8±8.1 | 0.740 |
| Albumin (mean±SD), g/dL | 4.8±4.9 | 4.1±0.4 | 0.323 | 3.9±0.4 | 3.8±0.5 | 0.483 |
| Creatinine (mean±SD), mg/dL | 1.0±0.8 | 1.0±0.4 | 0.603 | 1.3±1.0 | 1.0±0.5 | 0.734 |
| eGFR<60 (mean±SD), mL/min/1.73 m^2^ | 60.1±21.7 | 61.4±19.3 | 0.220 | 57.7±29.2 | 57.9±21.7 | 0.977 |
| Total cholesterol^b^ (mean±SD), mg/dL | 173.9±37.5 | 172.3±37.5 | 0.735 | 155.5±32.2 | 157.7±36.0 | 0.705 |
| Triglycerides (mean±SD), mg/dL | 111.8±58.3 | 117.4±70.3 | 0.792 | 103.1±51.0 | 92.9±51.8 | 0.406 |
| C-reactive protein^b^ (mean±SD), mg/dL | 0.4±1.1 | 0.4±0.8 | 0.620 | 0.2±0.2 | 0.5±0.9 | 0.121 |
| Hemoglobin (mean±SD), g/dL | 13.4±1.5 | 12.8±2.3 | 0.325 | 12.14±2.2 | 11.5±1.3 | 0.063 |
| BNP (mean±SD), pg/mL | 202.2±270.8 | 238.2±252.8 | 0.236 | 497.8±521.0 | 400.6±454.7 | 0.577 |
| Hemoglobin A1c^b^ (mean±SD), % | 5.9±1.1 | 6.2±0.8 | 0.107 | 6.3±1.5 | 6.5±1.4 | 0.649 |
| Transthoracic echocardiography |  |  |  |  |  |  |
| Left ventricular ejection fraction (mean±SD), % | 60.9±12.3 | 58.2±12.3 | 0.471 | 52.2±12.6 | 57.6±12.4 | 0.173 |
| Type of surgery for cardiovascular disease, n (%) |  |  |  |  |  |  |
| Valve replacement/repair | 36 (60.0) | 30（60.0) | 1.000 | 11 (73.3) | 31 (77.5) | 0.746 |
| CABG | 20 (33.3) | 20 (40.0) | 0.469 | 4 (26.7) | 18 (45.0) | 0.216 |
| Aorta replacement | 9 (15.0) | 9 (18.0) | 0.672 | 0 (0) | 2 (5.0) | 0.378 |
| Combined operation | 5 (8.3) | 9 (18.0) | 0.130 | 0 (0) | 11 (27.5) | **0.023** |
| Duration of surgery (mean±SD), min | 319.6±95.5 | 410.0±174.7 | **≤0.001** | 298.1±81.5 | 398.7±124.2 | **0.004** |
| CPB time (mean±SD), min | 161.4±56.0 | 212.9±88.4 | **0.001** | 144.9±40.5 | 197.5±82.9 | **0.020** |
| Aorta cross clamp time (mean±SD), min | 109.3±59.3 | 141.4±74.4 | **0.014** | 102.0±44.8 | 141.3±77.4 | **0.036** |
| Intraoperative blood loss (mean±SD), mL | 1351.9±799.2 | 1631.2±1738.3 | 0.663 | 1166.5±589.8 | 1717.6±1113.1 | 0.070 |
| Transfusion of red blood cells (mean±SD), unit | 4.9±4.1 | 7.2±7.2 | 0.198 | 6.4±4.6 | 9.7±5.2 | **0.044** |
| Intubation time (mean±SD), hours | 9.5±9.1 | 36.6±77.4 | **0.022** | 13.7±23.3 | 32.8±60.7 | 0.094 |
| ICU stay (mean±SD), days | 1.6±2.0 | 2.6±3.8 | 0.108 | 1.5±1.4 | 3.0±3.5 | 0.081 |
| Time from after surgery to discharge (mean±SD), days | 14.9±2.7 | 32.9±28.6 | **≤0.001** | 15.9±2.6 | 39.3±42.4 | **≤0.001** |
| Discharge destination (home), n (%) | 58 (96.7) | 48 (96.0) | 0.852 | 13 (86.7) | 35 (87.5) | 0.934 |

SD: standard deviation; BMI: body mass index; eGFR: estimated glomerular filtration rate; BNP: brain natriuretic peptide; CABG: coronary artery bypass grafting; CPB: cardiopulmonary bypass; ICU: intensive care unit.

^a^Using the Chi-squared test or Mann-Whitney U test

^b^Missing values in SMI (n=6), gait speed (n=19), handgrip strength (rt) (n=4), total cholesterol (n=1), and hemoglobin A1c (n=1) were excluded.

Supplementary Table 4 Factors associated with long-term hospitalization in patients with sarcopenia (excluding hemodialysis patients)

| Variables | OR | 95% CI | | | P-value^a^ |
| --- | --- | --- | --- | --- | --- |
| Albumin | 0.237 | 0.067 | - | 0.844 | **0.026** |
| C-reactive protein | 5.190 | 0.843 | - | 31.964 | 0.076 |
| CPB time | 1.015 | 1.003 | - | 1.027 | **0.012** |
| ICU stay | 1.330 | 0.981 | - | 1.803 | 0.066 |

The sarcopenia group was divided into two groups based on the median time from after surgery to discharge (25 days).

^a^Using a univariate logistic regression analysis including variables with P<0.05 in the Chi-square test or Mann-Whitney U test. OR: odds ratio; 95% CI: 95% confidence interval.

CPB: cardiopulmonary bypass; ICU: intensive care unit.

Supplementary Table 5 Independent factors of prolonged postoperative hospitalization in patients with sarcopenia in the multivariable analysis (excluding hemodialysis patients)

A: Model 1

| Independent variable | Odds ratio | 95% Confidence interval | | | P-value^a^ |
| --- | --- | --- | --- | --- | --- |
|  |  | Lower limit |  | Upper limit |  |
| Sex^b^ | 1.626 | 0.494 | - | 5.351 | 0.424 |
| Age^b^ | 1.033 | 0.964 | - | 1.107 | 0.352 |
| CPB time | 1.016 | 1.004 | - | 1.029 | **0.008** |

^a^Using multivariate logistic regression analysis adjusted for CPB time.

^b^Adjusted for sex and age.

CPB: cardiopulmonary bypass

B: Model 2

| Independent variable | Odds ratio | 95% Confidence interval | | | P-value^a^ |
| --- | --- | --- | --- | --- | --- |
|  |  | Lower limit |  | Upper limit |  |
| Sex^b^ | 1.427 | 0.382 | - | 5.326 | 0.597 |
| Age^b^ | 1.023 | 0.941 | - | 1.112 | 0.596 |
| CPB time | 1.012 | 1.000 | - | 1.024 | **0.048** |
| Albumin | 0.373 | 0.081 | - | 1.717 | 0.206 |
| C-reactive protein | 2.931 | 0.339 | - | 25.309 | 0.328 |
| ICU stay | 1.254 | 0.899 | - | 1.749 | 0.182 |

Duration of surgery was excluded because it included CPB time and aorta cross clamp time.

^a^Using a multivariate logistic regression analysis with variables with P<0.1 from Supplemental Table 4.

^b^Adjusted for sex and age.

CPB: cardiopulmonary bypass; ICU: intensive care unit
